# Impedance Spectrum in Cortical Tissue: Implications for Propagation of LFP Signals on the Microscopic Level

**DOI:** 10.1523/ENEURO.0291-16.2016

**Published:** 2017-01-31

**Authors:** Stéphanie Miceli, Torbjørn V. Ness, Gaute T. Einevoll, Dirk Schubert

**Affiliations:** 1 Department of Cognitive Neuroscience, Donders Institute for Brain, Cognition and Behaviour, Radboud University Medical Centre Nijmegen, 6500 HB, Nijmegen, The Netherlands; 2 Department of Mathematical Sciences and Technology, Norwegian University of Life Sciences, 1432 ÅS, Norway; 3 Department of Physics, University of Oslo, 0316 Oslo, Norway; 4 Department of Neural Networks, Center of Advanced European Studies and Research (caesar), Max Planck Society

**Keywords:** conductivity, cortex, local field potential, multielectrode array, neuronal tissue, signal frequency

## Abstract

Brain research investigating electrical activity within neural tissue is producing an increasing amount of physiological data including local field potentials (LFPs) obtained via extracellular *in vivo* and *in vitro* recordings. In order to correctly interpret such electrophysiological data, it is vital to adequately understand the electrical properties of neural tissue itself. An ongoing controversy in the field of neuroscience is whether such frequency-dependent effects bias LFP recordings and affect the proper interpretation of the signal. On macroscopic scales and with large injected currents, previous studies have found various grades of frequency dependence of cortical tissue, ranging from negligible to strong, within the frequency band typically considered relevant for neuroscience (less than a few thousand hertz). Here, we performed a detailed investigation of the frequency dependence of the conductivity within cortical tissue at microscopic distances using small current amplitudes within the typical (neuro)physiological micrometer and sub-nanoampere range. We investigated the propagation of LFPs, induced by extracellular electrical current injections via patch-pipettes, in acute rat brain slice preparations containing the somatosensory cortex *in vitro* using multielectrode arrays. Based on our data, we determined the cortical tissue conductivity over a 100-fold increase in signal frequency (5–500 Hz). Our results imply at most very weak frequency-dependent effects within the frequency range of physiological LFPs. Using biophysical modeling, we estimated the impact of different putative impedance spectra. Our results indicate that frequency dependencies of the order measured here and in most other studies have negligible impact on the typical analysis and modeling of LFP signals from extracellular brain recordings.

## Visual Abstract

**Figure:**
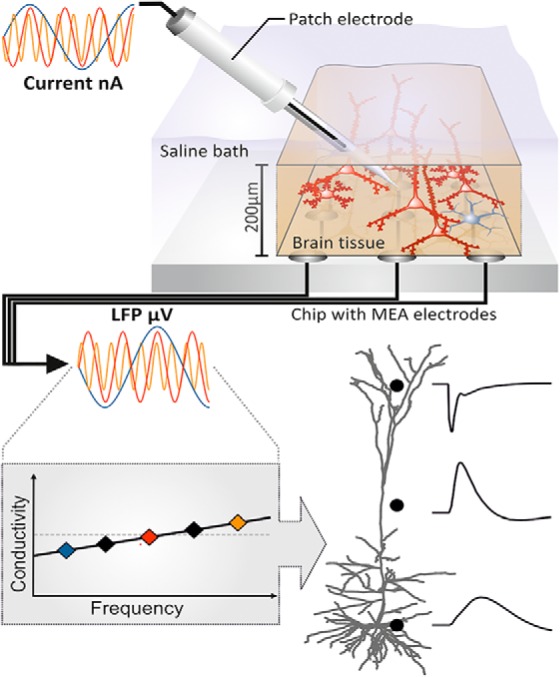

## Significance Statement

In order to unravel the mechanisms underlying the function or dysfunction of the healthy and diseased brain, researchers perform electrophysiological *in vivo* studies in various species for investigating neuronal activity. Recorded extracellular signals, like EEG, electrocorticography, or local field potentials, have propagated through neural tissue from the underlying neural sources to the respective recording electrodes. Consequently, a correct interpretation of the recorded signals relies on knowledge of whether the intrinsic biophysical properties of the tissue and its extracellular medium bias the recorded signals for example with respect to its frequency content. Our results, based on experimental *ex vivo* data and modeling, demonstrate a negligible bias for the propagation of neuronal signals through cortical tissue with respect to neurophysiologically relevant frequencies.

## Introduction

With the rapid development of multielectrodes with tens, hundreds, or thousands of electrode contacts, the use of *in vivo* and *in vitro* extracellular recordings of neural activity experiences a renaissance (Andersen et al., 2004; Buzsáki, 2004; Buzsáki et al., 2012; Einevoll et al., 2013b). Accurate and reliable interpretation of the neuronal signals requires a thorough understanding of the electrical properties of the underlying brain tissue (Nunez and Srinivasan, 2006; Einevoll et al., 2013b). One particularly pertinent question is whether the extracellular electrical conductivity of brain tissue is frequency dependent and thus biases the recorded electrophysiological signals (Gilja and Moore, 2007).

The extracellular medium of the brain consists of tightly packed cell membranes embedded in cerebrospinal fluid (CSF) (for review, see Syková and Nicholson, 2008). For frequencies relevant for neural recordings (i.e., less than a few thousand hertz), the cellular membranes of neurons and glial cells are expected to be largely nonconducting, so that currents can easily pass around them through the more conductive CSF (Nicholson and Syková, 1998; Peters et al., 2001; Pettersen et al., 2012; Nelson et al., 2013). If so, it may be expected that the frequency-independent conductivity of the CSF would translate into largely frequency-independent conductivity of the extracellular brain tissue.

Earlier studies (Nicholson and Freeman, 1975) indeed found such frequency independence. However, a later study (Gabriel et al., 1996) suggested a strong frequency-dependent increase of tissue conductivity for frequencies <100 Hz, in the range of physiological local field potentials (LFPs). Such a frequency dependence would bias recorded LFP signals toward lower frequencies. However, the study by Gabriel et al. (1996) used a two-electrode setup, where electrodes are used both for current injection and the measurement of extracellular potentials, which can be sensitive to electrode polarization (EP) and potentially have large effects on the measured conductivity (Gabriel et al., 1996; Mirtaheri et al., 2005; Nelson et al., 2008; Ishai et al., 2013).

In contradiction to these findings, more recently Logothetis et al. (2007) used a four-electrode setup, using separate electrodes for injection and measurement, eliminating the electrode polarization artifact (Mirtaheri et al., 2005; Logothetis et al., 2007; Goto et al., 2010; Ishai et al., 2013; Wagner et al., 2014). This study observed a negligible frequency dependence of the conductivity *in vivo* with frequencies ranging between 10 and 5000 Hz (Logothetis et al., 2007). Yet, in this study the distance between the electrodes was 3 mm, and test currents within the microampere range, larger than the sub-nanoampere currents typically passing through membranes of neurons in *in vivo* conditions. It was later argued that these high current amplitudes could mask a real frequency dependence of the extracellular conductivity within local brain microcircuits when recording LFPs in an *in vivo* situation (Bédard and Destexhe, 2009).

In the present *in vitro* study, we investigated within the rodent primary somatosensory (barrel) cortex, the frequency dependence of electrical properties of brain tissue at the microscopic level, both in terms of distances between current source (mimicking a genuine neural source of activity) and recording electrode (∼100 μm) as well as in terms of the current amplitudes (0.1–0.5 nA). Our experimental results revealed, at most, a weak frequency dependence of the electrical conductivity. In order to estimate the consequences of a frequency dependence of this order, we investigated the impact of different putative impedance spectra using biophysical modeling. We demonstrate that the frequency dependencies reported here will have negligible impact on the propagation of neuronal signals through cortical tissue.

## Materials and Methods

### Experimental methods

All animals were bred and reared in the Central Animal Laboratory of the Radboud University Nijmegen Medical Centre (Nijmegen, The Netherlands). Animals were supplied with food and water *ad libitum* and were kept on a 12 h dark/light cycle (lights on at 6:00 A.M.). All experiments were approved by the Committee for Animal Experiments of the Radboud University Nijmegen Medical Centre, Nijmegen, The Netherlands (Ru-DEC 2014-046), and all efforts were made to minimize animal suffering and to reduce the number of animals used.

#### Acute brain slice preparation

Brain slices were obtained from male juvenile (postnatal day 21–25) Wistar rats. Following decapitation performed under anesthesia, the brain tissue containing the barrel cortex was excised, quickly removed from the skull, and transferred to ice-cold cutting-and-storage artificial CSF (ACSF) oxygenated with carbogen (95% O_2_, 5% CO_2_). Cutting-and-storage ACSF consisted of the following (in mm): 124 NaCl, 1.25 NaH_2_PO_4_, 26 NaHCO_3_, 1 CaCl_2_, 5 MgCl_2_, 3 KCl, and 10 glucose at pH 7.4. Afterward, thalamocortical brain slices of 200 µm thickness were produced following a protocol described by Land and Kandler (2002) in ice-cold carbogenated ACSF using a vibratome (Microm HM 650 V, Microm). The brain slices were collected and stored in an incubation chamber containing carbogenated ACSF at room temperature for at least 1 h.

#### Multielectrode array recording

For electrophysiological recordings, we transferred the slices individually to the multielectrode array (MEA) recording chamber (standard 60 electrodes MEA chip, 60MEA200/30iR-Ti-gr, MCS; RRID:SCR_014809) under submerged conditions. The recording chamber and the attached MEA amplifier system (MEA1060-Up amplifier, MCS) was mounted on a fixed-stage upright microscope (BX51WI, Olympus Europe) and attached with an IR-sensitive CCD video camera system (DAGE IR-100, DAGE-MTI). In the recording chamber, the slices were continuously superfused at room temperature with carbogenated recording ACSF (ACSF_R_; in mm: 124 NaCl, 1.25 NaH_2_PO_4_, 26 NaHCO_3_, 2 CaCl_2_, 1 MgCl_2_, 3 KCl, and 10 glucose at pH 7.4; 0.5 ml/min). Under low magnification (fourfold), we placed the brain slices in such a way that somatosensory cortical layers were aligned with the electrode rows, whereas electrode row number 4 was always positioned underneath the barrels in cortical layer IV. Consequently, barrel-associated cortical columns were vertically aligned with respective columns of electrodes of the chip. Slices were held in position by Teflon-coated harp grids (ALA) and were left a minimum of 30 min before the onset of electrophysiological recording in order to ensure proper contact between electrodes and the cortical tissue. Electrical activities were simultaneously recorded with 60 substrate-embedded titanium nitride electrodes with 30 µm diameters and 200 µm spacing, and arranged in an 8 × 8 matrix. After 1200× amplification (single-ended amplifier, bandwidth 0.1 Hz to 3 kHz), signals were sampled at a rate of 20 kHz using a commercial data acquisition system (MCRack, MCS) and further analyzed.

#### Current injection protocol

**Figure 1.**
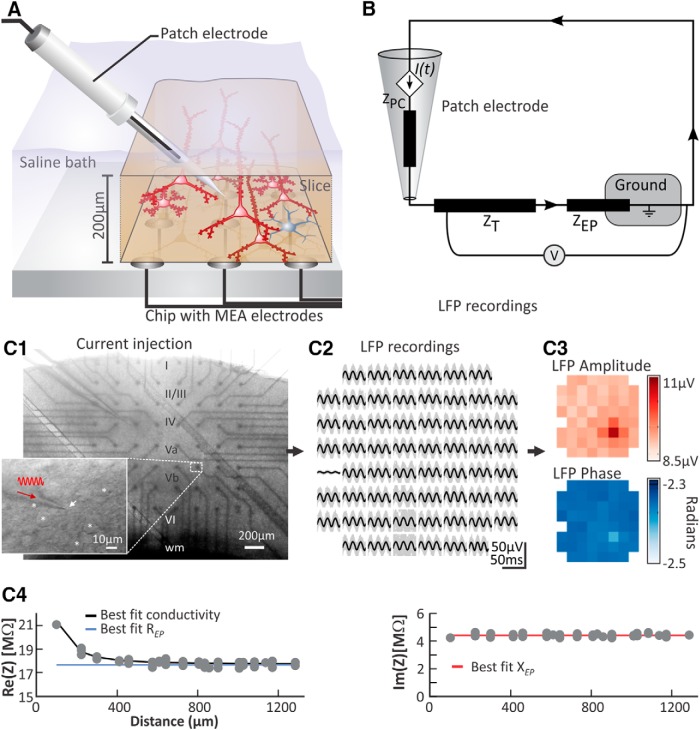
Experimental setup. ***A***, Current was injected by a patch electrode into the extracellular medium of a 200-µm-thick thalamocortical slice preparation immersed in ACSF (excitatory cells are represented in red, and inhibitory cells in gray). The extracellular potential was measured simultaneously by all electrodes of the MEA beneath. ***B***, The recording setup can be represented as an equivalent circuit where current flows from the patch-clamp electrode toward the ground, while the electric potential is recorded by a voltmeter. *Z*_PC_, Patch-clamp impedance; Z_T_, tissue impedance; V, voltmeter (MEA electrodes); Z_EP_, electrode polarization impedance. ***C***, Schematic overview of the recording and analysis routine. ***C1***, Picture photograph of the acute brain slice preparation during the current injection and LFP recording. Roman numbers indicate the position of layers I to VI of the somatosensory cortex. wm, White matter. Enlarged section zooming in on layer Vb shows neuron somata (asterisks), the positioning of the tip of the patch pipette (white arrow) and an illustration of the injection of sinusoidal current (red) into the cortical tissue. ***C2***, LFP recording via the MEA. The array shows for each electrode the 50 recorded sweeps (gray) and, superimposed, the average LFP (black). ***C3***, The LFP amplitude and phase at a given current injection frequency was extracted from the averaged LFPs using a Fast Fourier Transformation. The largest LFP amplitude was detected at the MEA electrode directly underneath the tip of the patch pipette. ***C4***, With the amplitude and phase of the LFP and injected current, we can find the total impedance, *Z*(**r**) = *Z*_T_(**r**) + *Z*_EP_ = *φ*_0_(**r**)/*I*_0_ e^(^*^j^* ^(^*^β^*^(^**^r^**^)-^ *^α^*^))^ (Eq. 8). The real part of *Z* (Re(*Z*); ***C4***, left) shows a decay with distance toward a constant, from which we can find the tissue conductivity (black line, Eq. 2) and *R*_EP_ (blue line). The imaginary part of *Z* is constant (***C4***, right), implying Im(*Z*) ∼ *X*_EP_ (red line), and Im(*Z*_T_) ∼ 0.

Borosilate glass pipettes (patch pipettes) with a tip resistance of 4–6 MΩ were filled with ACSF_R_ solution and used for current injection either into brain tissue or into ACSF bath solution. Control ACSF (ACSF_C_; in mm: 31 NaCl, 0.3 NaH_2_PO_4_, 6.5 NaHCO_3_, 0.5 CaCl_2_, 0.25 MgCl_2_, 0.75 KCl, and 2.5 glucose at pH 7.4) contained one-fourth of the NaCl concentration of ACSF_R_ and was used for current injection into bath solution only. Under visual control at 40× magnification (40×/0.75 W; Olympus) using infrared-enhanced quarter field illumination for contrast enhancement (DGC, Luigs & Neumann), we inserted the patch pipettes into layer Vb of the primary somatosensory cortex. A motorized biomanipulator and microscope stage system (SM7, Luigs & Neumann), which was coupled to a microscope control software system (Morgentau M1, Morgentau Solutions), allowed us to exactly position the patch pipette in three dimensions, in respect to the depth, the cortical layers, and the MEA electrodes. Within the tissue, for each experiment the tip of the pipette was positioned at two different depths in order to allow current injections at two different distances from the closest recording electrode in the MEA chip (distances of 100 and 125 µm). We took care to position the electrode within the extracellular matrix as far away as possible from any neighboring neuronal somata (i.e., typically at distances of ∼20 µm; Fig. 1*C1*). For the ACSF control experiments, the patch pipette was positioned at the same distance to the MEA electrodes as for the tissue experiment. Oscillatory current of varying amplitude (175, 300, and 500 pA maximum deflection) and frequency (5, 60, 100, 300, and 500 Hz) were injected through the patch pipette (SEC-05L, npi). The range of current amplitudes was chosen because it represents a range of single-neuron transmembrane currents as they occur physiologically during strong subthreshold or suprathreshold activity. Excitatory neurons in layers Va and Vb of the rat somatosensory cortex can be expected to have input resistances at ∼120 MΩ and membrane potentials of approximately −70 mV after corrections for liquid junction potentials of ∼10 mV (Schubert et al., 2001, 2006). A transmembrane current in the range of 175 pA would therefore be associated with a depolarization of 15–20 mV and, thus, would rarely induce action potential firing, whereas a current in the range of 500 pA can be expected to induce robust spiking.

### Theoretical background

#### Volume conductor theory

Transmembrane currents give rise to extracellular potentials (Nunez and Srinivasan, 2006). In the commonly used volume conductor theory the electrical properties of a medium (e.g., brain tissue) are described in terms of an electrical conductivity, σ, which in principle can be a scalar or a tensor, real or complex (Pettersen et al., 2012). In an infinite brain tissue volume conductor where the extracellular conductivity *σ*_T_ of the brain tissue is assumed to be real (ohmic), isotropic (same in all directions), and homogeneous (same at all positions), the fundamental formula giving the contribution from a transmembrane current *I*(*t*) at position (*x'*, *y'*, *z'*) to the extracellular potential *ϕ_h_*(*x*, *y*, *z*, *t*) in the brain tissue at a position (*x*, *y*, *z*) is given by (Holt and Koch, 1999; Pettersen et al., 2012; Lindén et al., 2014),

(1) $$\phi_{h}(x,y,z,t)=\frac{1}{4\pi \sigma_{T}}\frac{I(t)}{\sqrt{{\left(x-x^{\prime }\right)}^{2}+{\left(y-y^{\prime }\right)}^{2}+{\left(z-z^{\prime }\right)}^{2}}}$$

Here the potential is assumed to be measured by an (ideal) extracellular point electrode, and the electrical ground (zero potential) is set to be infinitely far away. For a thorough review of its derivation, assumptions, and limitations, see Hämäläinen et al. (1993), Nunez and Srinivasan (2006), and Pettersen et al. (2012).

The present MEA setup does not correspond to an infinite homogeneous volume conductor, but by using the method of images, Equation 1 can be extended to also be applicable for *in vitro* slice MEA measurements, where one has a three-layered medium (nonconducting MEA plate, brain tissue slice, and ACSF bath; Ness et al., 2015; Fig. 1*A*). With a single current point source, *I*(*t*), positioned at (*x'*, *y'*, *z'*) in the middle slab of a three-layered medium (i.e., a lower, nonconducting glass electrode plate, an electrically homogeneous brain tissue slice with vertical extension from *z =* 0 to *z = h*, and an infinitely thick ACSF layer covering the brain slice), the extracellular potential at the electrode–slice boundary (*z =* 0) is given by Ness et al. (2015) as follows:

(2) $$\phi_{MEA}(x,y,0,t)=2\phi_{h}(x,y,0,t)+2\sum_{n=1}^{\infty }{\left(\frac{\sigma_{T}-\sigma_{S}}{\sigma_{T}+\sigma_{S}}\right)}^{n}\left(\phi_{h}(x,y,2nh,t)+\phi_{h}(x,y,-2nh,t)\right).$$

Here *ϕ_h_* is given by Equation 1, and *σ*_S_ is the electrical conductivity of the ACSF. Note that for *σ*_T_ = *σ*_S_, this reduces to give twice the size of the potential predicted from the homogeneous point source Equation 1. This is as expected for a source positioned in a semi-infinite half-space above a semi-infinite nonconducting medium (Jackson, 1998; Ness et al., 2015). The expression contains an infinite sum, but in practice the sum converges fast when terms are added. When evaluating the expression, we have here truncated the series at 20 terms, which gives sufficient accuracy (Ness et al., 2015). In the present study, the current source *I*(*t*) corresponds to a current injection into the extracellular part of the brain tissue (Fig. 1*B*); however, the formalism is equally valid when the current source stems from transmembrane currents (Lindén et al., 2014).

#### Correction for electrode polarization

EP originates at the electrolyte–metal interfaces of current carrying electrodes and can severely complicate conductivity estimation (Schwan, 1992; Gabriel et al., 1996; Martinsen and Grimnes, 2008). The procedure followed for EP correction by Gabriel et al. (1996) and others (Schwan, 1992; Mirtaheri et al., 2005; Ishai et al., 2013; Wagner et al., 2014) was to first estimate the effect of EP in ACSF measurements where the salt content of ACSF_C_ was scaled down to have a similar conductivity as brain tissue. Since the conductivity of ACSF should be frequency independent, any frequency dependence observed in the experimentally measured ACSF conductivity is assumed to be due to electrode polarization. In our experimental setup, current is injected into saline or neural tissue by a patch-clamp electrode and flows to ground, which is a silver ball within the saline bath located at a distance of 15 mm from the patch-clamp electrode and of 10 mm from the closest recording electrode on the MEA chip. Since EP is assumed to stem from the electrolyte–metal interface of current-carrying electrodes, and since we are measuring the potential relative to ground for a fixed current source, we reckon that the electrode polarization occurs at the silver-ball ground electrode (Ø. Martinsen and H. Kalvøy, personal communication). This means that for a given frequency, the EP impedance (*Z*_EP_) should be the same for tissue measurements and saline measurements, since the electrolyte–metal interface at the silver ball is unaffected by the presence of the neural slice.

The equivalent electric circuit for our experimental setup is shown in Figure 1*B*. For a current *I*(*t*) injected into a saline bath (purely resistive; i.e., *σ*_S_ is real) at position ***r****' =* (*x'*, *y'*, *z'*) above an MEA electrode plate, the electric potential at the MEA plane at position ***r***= (*x*, *y*, *z*), relative to ground far away, can be written as follows:

(3) $$\phi (r,t)=\frac{1}{2\pi \sigma_{S}}\frac{I(t)}{\left|r-r\prime \right|}=Z_{S}(r)I(t).$$

where *Z*_s_ is the impedance (or resistance) of the saline medium, *σ_S_* is the conductivity of the medium, and |***r*** - ***r****'*| is the distance between the point source and the measurement point (Ness et al., 2015). For an injected sinusoidal current *I*(*t*) with an angular frequency *ω =* 2π*f*, phase *α*, and amplitude *I*_0_, we can write *I*(*t*) = *I*_0_e*^j^* ^(^*^ωt^* ^+^ *^α^* ^)^, with *j* being the imaginary unit. The recorded potentials will then also be sinusoidal, and for a resistive medium they will have the same phase as the input current. If we also have to consider a *Z*_EP_, it has to be added in series with *Z*_s_, giving the following expression for sinusoidal current:

(4) $$\phi (r,t)=(Z_{s}(r)+Z_{EP})I(t).$$

Note that this expression assumes sinusoidal input currents, but since an arbitrary signal can be represented as a sum of sinusoids, it can be extended to also cover more complicated cases (see Homogeneous frequency-dependent medium). The recorded potentials will also in this case be sinusoidal, $\phi (r,t)=\phi_{0}(r)e^{j(\omega t+\beta (r,\omega ))}$, where a phase shift might have been introduced from the EP so that β≠α. $\phi_{0}(r,\omega )$ and β(r,ω) are experimentally measured at each recording electrode. In the way the experimental protocol was implemented, the phase of the injected current, α(ω), relative to the measured potential was not available and was therefore obtained from current injections in saline (see below). For a given angular frequency, we can write Equation 4 as follows:

(5) $$\phi_{0}\left(r,\omega \right)e^{j\beta \left(r,\omega \right)}=\left(Z_{S}\left(r\right)+Z_{EP}\left(\omega \right)\right)I_{0}e^{j\alpha \left(\omega \right)}.$$

We know that for a given angular frequency, $Z_{EP}(\omega )=R_{EP}(\omega )+jX_{EP}(\omega )$ should be a constant (i.e., should be the same for all MEA recording electrodes), and it can therefore be estimated as follows:

(6) $$Z_{EP}(\omega )=average\left(\frac{\phi_{0}(r,\omega )}{I_{0}}e^{j\left(\beta (r,\omega )-\alpha (\omega )\right)}-\frac{1}{2\pi \sigma_{S}\left|r-r\prime \right|}\right)\,.$$

This can be used to minimize the following expression:

(7) $$\frac{\phi_{0}(r,\omega )}{I_{0}}e^{j\left(\beta (r,\omega )-\alpha (\omega )\right)}-\frac{1}{2\pi \sigma_{S}\left|r-r\prime \right|}-Z_{EP}(\omega )=0,$$

to obtain estimates of the conductivity *σ_S_* and input current phase *α* for each frequency. An internal consistency check is that the imaginary part of $\frac{\phi_{0}(r,\omega )}{I_{0}}e^{j\left(\beta (r,\omega )-\alpha (\omega )\right)}$, corresponding to *X*_EP_, should show no dependence with position, since saline is a purely ohmic medium with no imaginary component of the impedance.

The impedance in the neural tissue recordings can be written as follows:

(8) $$Z_{T}(r,\omega )=\frac{\phi_{0}(r,\omega )}{I_{0}}e^{j\left(\beta (r,\omega )-\alpha (\omega )\right)}-Z_{EP}(\omega ),$$

where it is not *a priori* known whether $Z_{T}(r,\omega )$ will have a significant imaginary part (i.e., exhibit any capacitive properties). We can, however, assume that both the imaginary and real parts of $Z_{T}(r,\omega )$ should be approximately proportional to 1/|***r***| (see Homogeneous frequency-dependent medium), and, thus, if the imaginary part of $Z_{T}(r,\omega )$ is found to be independent of ***r***, this would imply negligible capacitive properties and resistive neural tissue. If so, one can estimate the conductivity *σ*_T_ of the tissue using Equation 2. A fitting procedure was implemented to estimate the conductivity of the slice and the EP based on this equation and the experimental data, assuming a constant conductivity, *σ*_S_, for the ACSF_R_ of 1.5 S/m. This value was estimated from current injections in ACSF_R_ at 500 Hz and is in full agreement with previously reported values (Nunez and Srinivasan, 2006; Logothetis et al., 2007).

#### Homogeneous frequency-dependent medium

It has been suggested that the electrical conductivity is frequency dependent also in the frequency range relevant for extracellular recordings in the brain (i.e., less than a few kilohertz; Bédard and Destexhe, 2009). To obey causality [i.e., that an extracellular potential ϕ(t) originating from a transmembrane current *I*(*t*) does not occur prior to the onset of the current], a frequency-dependent conductivity will require that it is complex i.e., $\overset{˜}{\sigma }(f)=\sigma_{R}(f)+j\sigma_{I}(f)$; Toll, 1956; Plonsey and Heppner, 1967; Orfanidis, 2004]. Here $\sigma_{I}(f)=2\pi f\epsilon (f)$ (Martinsen and Grimnes, 2008), where ϵ(f) is the permittivity of the medium. In polar form, this can be expressed as $\overset{˜}{\sigma }(f)=\left|\overset{˜}{\sigma }(f)\right|e^{j\theta (f)}$, where $\theta (f)=arctan\left(\sigma_{I}(f)/\sigma_{R}(f)\right)$. In this case, Equation 1 generalizes to the following:

(9) $$\phi_{h}\left(r,t\right)=\overset{\infty }{\underset{-\infty }{\int }}\frac{1}{4\pi \left(\sigma_{R}\left(f\right)+j\sigma_{I}\left(f\right)\right)}\frac{I\left(f\right)e^{j2\pi ft}}{\left|r-r\prime \right|}df=\overset{\infty }{\underset{-\infty }{\int }}\frac{e^{-j\theta \left(f\right)}}{4\pi \left|\overset{˜}{\sigma }\left(f\right)\right|}\frac{I\left(f\right)e^{j2\pi ft}}{\left|r-r�\right|}df$$

where *I*(*f*) is the Fourier-transformed current given by *I*(*f*) = ∫_∞_ *I*(*t*) e^−^*^j^* ^2π^*^ft^dt*.

Causality requires a particular relationship between the real part ($\sigma_{R}(f)$) and imaginary part ($\sigma_{I}(f)=2\pi f\epsilon (f)$) of the conductivity, or, equivalently, between the frequency response of the magnitude, |$\overset{˜}{\sigma }(f)$|, and the phase shift, *θ*(*f*), called the Bode relation (Clark, 2004; Warwick, 2010; Bechhoefer, 2011). The phase shifts, *θ*(*f*), can under these assumptions be reconstructed from an experimentally measured $\left|\overset{˜}{\sigma }(f)\right|$, by using the cepstrum method. For implementational details, see the study by Clark (2004). Having obtained the phase shifts *θ*, the guaranteed causal extracellular potentials were calculated using Python’s Scipy.fftpack, as follows:

(10) ϕh(r,t)=Re⁡(iFFT[e−jθ(f)I(f)4π|σ˜(f)||r−r′|])

where I(f)=FFT[I(t)] is the current transformed to the frequency domain by a fast Fourier transform (FFT), and Re means that we take the real part of the expression. This approach lets us link a measured magnitude of the conductivity, $\left|\overset{˜}{\sigma }(f)\right|$, to the phase shift, *θ*(*f*); notice, however, that it requires full knowledge of the magnitude response over the entire frequency spectrum. In this study, we interpolate and extrapolate based on sparse data, and, thus, our resulting complex conductivity should not be considered more than a plausible approximation.

#### Data analysis

For each recording, 50 sweeps of 2.5 s were averaged over the 60 electrodes of the MEA chip, and the signal amplitude and phase were extracted at the stimulated frequency using a FFT. An illustration summarizing our approach is given in Figure 1*C*. Statistical analysis was performed using multivariate ANOVA with *post hoc* pairwise comparisons (Bonferroni corrected), paired two-tailed Student’s *t* test, and linear regression analysis (SPSS version 9, SPSS). Data are presented as individual recordings or as the mean ± SEM. For all tissue and ACSF recordings, we marked recording electrodes that showed strongly reduced responses (compared with responses of the neighboring electrode) and removed them from further analysis.

#### Cross-validation

For cross-validation, we repeated all of the analysis using only half the experimental data, either using odd-numbered sweeps or even numbered sweeps. This led to very minor adjustments of the individual estimated conductivities and had no significant effect on the analysis.

#### Method validation

To test the robustness of our conductivity estimation procedure, we made model-based test data with similar noise characteristics as those found within the experimental data. The forward modeling was based on Equation 2 and was stored in the same form as the experimental data. Even for very high noise levels, our method allowed an unbiased estimation of both the electrode polarization and the extracellular conductivity for all tested injection positions within the slice, as well as for different slice thicknesses. We also confirmed that small errors in the localization of the current injection, or the conductivity of ACSF_R_, only changed the overall magnitude of the conductivities and not the frequency dependence. Furthermore, we produced anisotropic test data, based on an anisotropic version of Equation 2 (Ness et al., 2015), to determine how sensitive our analysis was to structural anisotropies of the tissue, such as the vertically aligned organization of apical dendrites in the cortex.

#### Computational modeling

To estimate the effect of a frequency-dependent conductivity on measured extracellular potentials, we show the effect on the extracellular potential from a single-cell model based on the experimentally constrained layer Vb cortical pyramidal cell model (Hay et al., 2011). The cell model was given synaptic input, modeled as an exponentially decaying conductance using ExpSyn in NEURON, in one case arriving at the soma strong enough to elicit a spike and in another case arriving at the apical dendrite not strong enough to elicit a spike. The synaptic input arrived 20 ms after the onset of the virtual recording, with a decay constant of 2 ms and a weight of 0.01 and 0.05 µS, respectively, for the nonspiking and spiking cases. In a third case, we used white noise current input, similar to Lindén et al. (2010) and Ness et al. (2016), i.e., a sum of sinusoids of equal amplitude, but random phases. The input current was scaled to have an SD of 8 pA, resulting in voltage fluctuations in the soma with an SD of 0.8 mV. The neural simulations had a time step of 1/32 ms, and the first 1000 ms of the simulation was discarded to make sure the cell was in its resting state. The resting membrane potential of the cell was set uniformly to −70 mV in both cases by shifting the passive leak reversal potential of the cell prior to the simulation onset. The calculation of the extracellular potentials assumed that the cell was embedded in an infinite, homogeneous, isotropic, but frequency-dependent medium (Eq. 10), and the extracellular potentials were calculated at three representative virtual electrode points at different heights along the axis of the main apical dendrite, corresponding to 0, 500, and 1000 µm relative to the soma. The distance from the axis of the main apical dendrite was 50 µm away for all three electrodes.

All neural simulations were performed with LFPy (RRID:SRC_014805; Lindén et al., 2014), a Python package with an interface to NEURON (RRID:SCR_005393; Hines and Carnevale, 1997; Carnevale and Hines, 2006; Hines et al., 2009). All modeling and plotting was performed in Python (RRID:SCR_008394; Langtangen, 2012). For the interested reader, a Python package with the code to reproduce all result figures in this study and all experimental data will be available upon request.

## Results

To assess the frequency dependence of electrical conductivity within cortical tissue, we mimicked a genuine neural source of current by injecting sinusoidal currents into the extracellular medium of an acute cortical brain slice via a stimulation electrode (i.e., patch pipette; Fig. 1). In all experiments, we applied identical sets of sinusoidal current injection protocols, consisting of five consecutive frequencies (5, 60, 100, 300, and 500 Hz), three amplitudes (maximum deflection, 175, 300, and 500 pA), and two different distances between the tip of the stimulation electrode and the nearest recording electrode of the MEA (125 and 100 µm). This allowed us to precisely estimate the brain tissue conductivity for each of the 30 arrangements (5 frequencies × 3 amplitudes × 2 distances), as illustrated in Figure 1*C* (also see Materials and Methods).

### Frequency-dependent effects in saline measurements

As a first step, in order to assess how the frequency of electrical signals affects their propagation within neural tissue, we determined the intrinsic frequency-dependent properties of the experimental setup. For frequencies less than ∼1 kHz (Schwan, 1992; Gabriel et al., 1996; Ishai et al., 2013), current-carrying electrodes are expected to be influenced by EP (Schwan, 1992; Gabriel et al., 1996; Ishai et al., 2013), which may substantially influence the measured conductivity of our experiments in the brain tissue. Since the conductivity of ACSF is known to be frequency independent for the tested frequency range (≤500 Hz; Gabriel et al., 1996, 2009; Nunez and Srinivasan, 2006; Wagner et al., 2014), we expected any frequency dependence observed here to be due to EP.

**Figure 2.**
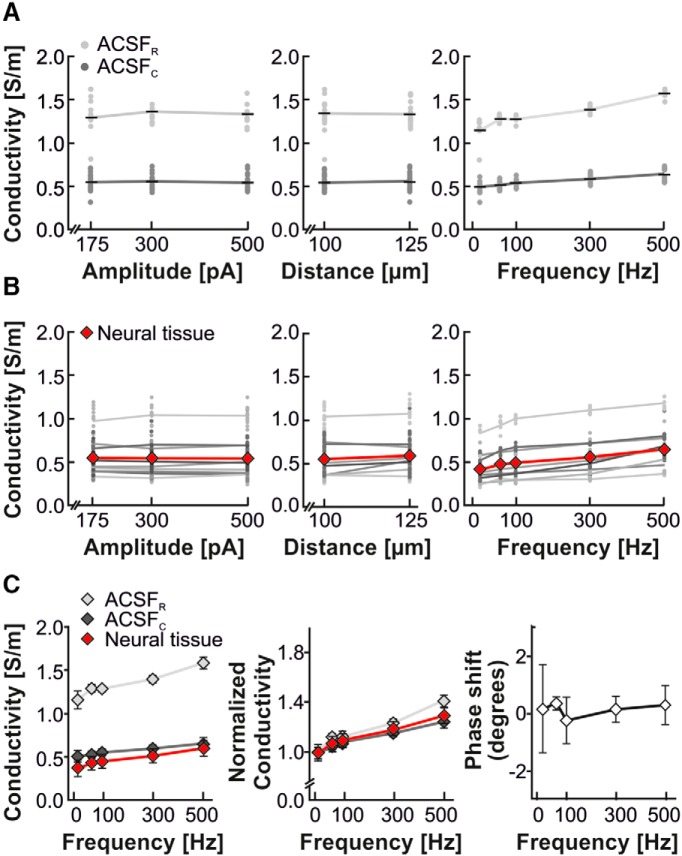
Current amplitude, distance, and frequency dependence of conductivity within ACSF and neural tissue. Conductivities were determined for different current amplitudes (175, 300, and 500 pA), distances between injection and closest recording electrode (100 and 125 µm), and current injections of different frequencies (5, 60, 100, 300, and 500 Hz). ***A***, conductivity (S/m) within ACSF (ACSF_R_, amplitude n = 2, distance n = 3, light gray) and control ACSF (ACSF_C_, amplitude n = 4, distance n = 6, black). Parameter-specific frequency dependence is shown for the pooled data of the remaining two parameters. Data are shown as the mean (diamonds) and individual data points (dots). ***B***, conductivity of neural tissue (amplitude n = 18, distance n = 27 recorded in 9 brain slices, mean is plotted in red). ***C***, Comparison of data recorded in ACSF and in cortical neural tissue based on data shown in ***A*** and ***B***. Conductivity as a function of frequency for ACSF_R_ (light gray), ACSF_C,_ (dark gray), and neural tissue (red) as absolute values (left) and normalized to the value at 5 Hz (middle). Right, Difference between phases measured in ACSF and neural tissue. Data are reported as the mean ± SEM.

To evaluate the effect of EP, we first injected a sinusoidal current following the protocol described above (five frequencies, three amplitudes, two distances), with a patch pipette positioned in ACSF only (i.e., no brain tissue in the recording chamber). EP is expected to occur at the electrode–electrolyte interface of current-carrying electrodes (current is injected at the patch-clamp electrode tip and leaves through the ground electrode). Placing the stimulation in standard ACSF_R_ alone resulted in generally high conductivities at ∼1.3–1.5 S/m (Fig. 2*A*, Table 1), which is more than two times as high as the expected conductivities in brain tissue superfused with ACSF_R_ (Goto et al., 2010; Fig. 2*B*). We addressed this by also testing EP in control ACSF_C_ containing one-fourth of the standard NaCl (31 mm NaCl; see Materials and Methods; Fig. 2*A*). In ACSF_C_, our experiments resulted in conductivities generally in the range of 0.5 S/m (Fig. 2*A*); therefore, similar to the expected conductivities in brain tissue (Logothetis et al., 2007).

**Table 1:** Conductivities in ACSF and brain tissue following different current injection conditions

ACSF_R_ conductivity (S/m)					
	Current amplitude, *n* = 6			Electrode distance, *n* = 6	
Frequency	175 pA	300 pA	500 pA	100 µm	125 µm
5 Hz	1.03 ± 0.02	1.24 ± 0.00	1.16 ± 0.00	1.07± 0.14	1.23 ± 0.03
60 Hz	1.29 ± 0.02	1.26 ± 0.03	1.27 ± 0.01	1.23 ± 0.01	1.27± 0.02
100 Hz	1.23 ± 0.05	1.30 ± 0.02	1.27 ± 0.01	1.29 ± 0.01	1.24 ± 0.03
300 Hz	1.36 ± 0.04	1.42 ± 0.02	1.39 ± 0.03	1.42 ± 0.01	1.36 ± 0.03
500 Hz	1.55 ± 0.06	1.60 ± 0.03	1.56 ± 0.00	1.60 ± 0.02	1.54± 0.03
ACSF_c_ conductivity (S/m)					
	Current amplitude, *n* = 12			Electrode distance, *n* = 12	
Frequency	175 pA	300 pA	500 pA	100 µm	125 µm
5Hz	0.46 ± 0.05	0.49 ± 0.04	0.48 ± 0.02	0.49 ± 0.03	0.46 ± 0.03
60Hz	0.52 ± 0.02	0.51 ± 0.02	0.50 ± 0.02	0.51 ± 0.02	0.52 ± 0.02
100Hz	0.55 ± 0.03	0.53 ± 0.02	0.53 ± 0.02	0.52 ± 0.02	0.55 ± 0.02
300Hz	0.59 ± 0.03	0.60 ± 0.03	0.59 ± 0.04	0.57 ± 0.03	0.61 ± 0.02
500Hz	0.63 ± 0.04	0.64 ± 0.05	0.63 ± 0.05	0.62 ± 0.03	0.65 ± 0.03
Brain slice conductivity (S/m)					
	Current amplitude, *n* = 54			Electrode distance, *n* = 54	
Frequency	175 pA	300 pA	500 pA	100 µm	125 µm
5 Hz	0.37 ± 0.03	0.37 ± 0.04	0.38 ± 0.04	0.37 ± 0.03	0.38 ± 0.03
60 Hz	0.40 ± 0.04	0.41 ± 0.04	0.42 ± 0.04	0.41 ± 0.04	0.41 ± 0.03
100 Hz	0.43 ± 0.05	0.42 ± 0.05	0.43 ± 0.05	0.42 ± 0.04	0.43 ± 0.04
300 Hz	0.48 ± 0.05	0.47 ± 0.05	0.47 ± 0.05	0.47 ± 0.04	0.48 ± 0.04
500 Hz	0.55 ± 0.05	0.54 ± 0.05	0.54 ± 0.05	0.53 ± 0.04	0.56 ± 0.04

Current amplitude and electrode distance do not affect the frequency-dependent increase in conductivity. Groups were compared using two-way ANOVA. Overall test statistics: ACSF_R_: amplitude, *F*_(8)_ = 0.45, *p* = 0.88; distance, *F*_(4)_ = 2.15, *p* = 0.11; ACSF_C_: amplitude, *F*_(8)_ = 0.06, *p* = 0.99; distance, *F*_(4)_ = 0.57, *p* = 0.68; brain slice amplitude: amplitude, *F*_(8)_ = 0.02, *p* = 1.00; distance, *F*_(4)_ = 0.14, *p* = 0.96.

For both ACSF_C_ and ACSF_R_, at all tested injection frequencies we found no significant effect of either the current amplitude or the distance between injection and recording electrodes on the conductivity (two-way ANOVA; current injection amplitude: ACSF_R_, *F*_(8)_ = 0.45, *p* **=** 0.88; ACSF_C_, *F*_(8)_ = 0.06, *p* = 0.99; electrode distance: ACSF_R_, *F*_(4)_ = 2.15, *p* = 0.11; ACSF_C_, *F*_(4)_ = 0.57, *p* = 0.68; Fig. 2*A*, Table 1). In contrast, we found a slight frequency-dependent increase in conductivity, especially for current injections into ACSF_R_. For ACSF_R_ from 5 to 500 Hz, conductivity increased by ∼50% (from on average 1.1 to 1.55 S/m; linear regression slope, 0.0007; *R* ^2^ = 0.72). For current injections into ACSF_C_, the frequency-dependent increase (33%; on average, 0.48–0.64 S/m from 5 to 500 Hz; linear regression slope, 0.0003; *R* ^2^ = 0.46) was significantly lower compared with ACSF_R_ (*F*_(1,58)_ = 26.55, *p* < 0.0001; Fig. 2*A*). Note that these values are corrected for EP (Eq. 7). As such, the source of this spurious frequency dependence of saline is unknown, although it must be intrinsic to the experimental equipment. The recordings in ACSF serve as a good estimate of the size of this effect: ∼50% increase in conductivity over a 100-fold increase in frequency. This estimate is important, because we must assume that the same spurious frequency dependence will be present in the actual recordings in neural tissue.

As neither current injection amplitude nor distance between injection and recording electrodes affected the recorded conductivity, in the following we pooled these measurements at each frequency (ACSF_R_, *n* = 6; ACSF_C_, *n* = 12) to further compare with conductivity measurements obtained in cortical tissue.

### Cortical tissue shows moderate frequency dependence similar to saline

In order to evaluate the intrinsic conductivity of cortical tissue and its possible frequency-dependent nature, we applied the current injection and recording protocols in *n* = 9 brain slice preparations, as previously described for current injection into ACSF only. We placed the stimulation electrode in the extracellular space between visually identified neurons in 200-µm-thick acute brain slice preparations containing the primary somatosensory (barrel) cortex (Fig. 1). There, we injected currents of different frequencies and amplitudes, and of varying distances from the closest recording electrode using the same protocol as described above for saline experiments. We decided upon thin slice preparations as it allowed us to optimally visualize the individual neurons in the vicinity of the patch pipette using infrared-enhanced imaging. By placing the patch electrode as far away from neuron somata and proximal dendrites as possible (∼20 µm distance; Fig. 1*C1*), we prevented our stimulation to activate intracellular signal propagation of nearby cells.

As a whole, the determined tissue conductivities showed values between 0.4 and 0.6 S/m (Fig. 2*B*, Table 1), and were, as expected, similar to the conductivities we found for ACSF_C_ (Fig. 2*C*). Similar to the results obtained in ACSF_R_ and ACSF_C_, for each of the tested frequencies increasing the amplitude or distance of the current injection had no significant effect on the tissue conductivity (two-way ANOVA; current injection amplitude: *F*_(8)_ = 0.02, *p* = 1.00, *n* = 18; electrode distance: *F*_(4)_ = 0.14, *p* = 0.97; *n* = 27 per frequency; Fig. 2*B*).

In cortical tissue, we observed a frequency-dependent linear increase of conductivity of ∼50% over the entire range of tested frequencies (linear regression slope, 0.0004; *R* ^2^ = 0.112). This frequency-dependent increase was a robust phenomenon observed in each of the individual experiments (Fig. 2*B*), and the frequency dependent increase found in cortical tissue was not significantly different than the frequency-dependent increase found in ACSF_C_ (*F*_(1,258)_ = 0.04, *p* = 0.84). Consequently, the conductivity in both saline and neural tissue showed no dependence on the current amplitude or distance between injection and recording site but showed an increase with frequency (Fig. 2*B*). When directly comparing the frequency dependence of the neural tissue conductivity with the corresponding results for ACSF_R_ and ACSF_C_, we found the frequency dependence to be very similar for saline and tissue (Fig. 2*C*). This similarity was also confirmed by comparing the trends of the conductivities normalized to the conductivity estimated at 5 Hz (Fig. 2*C*).

### Phase measurements imply lack of capacitive effects in cortical tissue

A capacitive extracellular medium would imply a conductivity described by a complex number, σ(f)→σ(f)+j2πfε(f) where *f* is the frequency, ε(*f*) is the permittivity of the tissue, and *j* marks the second term as imaginary (Hämäläinen et al., 1993; Nunez and Srinivasan, 2006; Pettersen et al., 2012). For a simple sinusoidal current injection, this would correspond to a phase shift between the injected current and the measured potential (Nunez and Srinivasan, 2006). However, in our experimental setup an observed phase shift between the injected current and recorded extracellular potential does not necessarily result from capacitive tissue, as the intrinsic properties of electrodes can also induce substantial phase shifts (Nelson et al., 2008; Ishai et al., 2013). To circumvent this complication, we compared the phases retrieved from recordings in either neural tissue or ACSF derived using identical current injection protocols. A phase difference here would imply capacitive effects in the neural tissue since no capacitive effects should occur in ACSF (Martinsen and Grimnes, 2008). We found no phase differences between recordings in saline (ACSF_R_ and ACSF_C_) and recording in neural tissue for any of the injected frequencies (Fig. 2*C***)**, indicating that any capacitive effects were negligible.

### Modeling implies negligible impact of moderate frequency dependence of tissue conductivity

**Figure 3.**
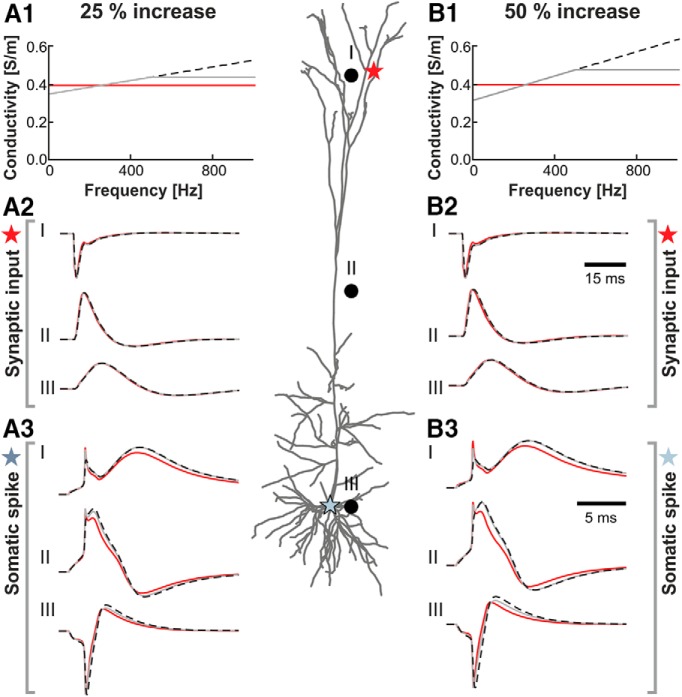
Simulated effect of frequency-dependent conductivity on extracellular potential arising from dendritic synaptic input as well as a somatic spike. Middle, Somatodendritic reconstruction of a layer V pyramidal neuron and three representative simulated extracellular recording electrodes (black dots; I–III). Extracellular recording of a synaptic input was simulated to take place at the level of the distal apical dendrite (red star), an action potential (spike) was simulated to be induced at the soma (blue star). ***A1***, Three different conductivity profiles, corresponding to a constant conductivity (red) or a linear increase in conductivity of 25% from 5 to 500 Hz that either stops increasing at 500 Hz (gray) or continues to rise linearly (black dashed). ***A2***, ***A3***, Normalized extracellular responses at electrodes I–III following the dendritic synaptic input (***A2***) or the somatic spike (***A3***) for the different conductivity profiles shown in ***A1***. ***B1***, Three different conductivity profiles similar to those in ***A1*** but for a 50% increase in conductivity. ***B2***, ***B3***, Normalized extracellular responses at electrodes I–III following the dendritic synaptic input (***B2***) or the somatic spike (***B3***) for the different conductivity profiles shown in ***B1***. Note that simulations of other extracellular recording positions led to different extracellular potentials but to a similar negligible impact of the frequency-dependent conductivity.

What implications does the experimentally determined impedance spectrum of cortical tissue have for the propagation of extracellular potentials? Using biophysical modeling, we calculated the extracellular potential using some predefined neural activity for different impedance spectra. This allowed us to precisely quantify the impact of a putative frequency-dependent conductivity compared with a fixed frequency-independent average value. As a model, we used a somatodendritic reconstruction of a pyramidal cell from cortical layer Vb (Hay et al., 2011). For technical reasons (i.e., limitations in the patch-clamp amplifier system), in the experiments we could not probe the frequency range >500 Hz, but in the modeling, for completeness, we tested the following two alternatives: (1) an increase in conductivity that stops at 500 Hz; and (2) one that continues to rise linearly (Fig. 3*A1*,*B1* ). Furthermore, we simulated the consequences of a 25% as well as a 50% frequency-dependent increase in tissue conductivity.

The LFP, the low-frequency part of the recorded cortical extracellular potential, is *in vivo* thought to be dominated by synaptic currents and their associated dendritic and somatic return currents (Einevoll et al., 2013a). We found that the extracellular potential generated by a single synaptic input to the apical dendrite of a pyramidal cell was little affected by the presently observed frequency dependence of the cortical conductivity (Fig. 3*A2*,*B2* ). We observed that even a 50% increase in conductivity from 5 to 500 Hz had a negligible effect. Since signals from synaptic input have very little signal power >500 Hz, these values were not affected by the assumption made for the conductivity >500 Hz.

For a spike, the impact on the extracellular potentials of a frequency-dependent conductivity in the frequency range observed here is seen to be modest (Fig. 3*A3*,*B3*), although more prominent than for the situation with synaptic input. For the spike recorded in the extracellular space close to the soma, the peak-to-peak amplitude for the average conductivity was 33.1 µV, which was reduced to 31.7 µV for a 25% increase in conductivity and to 31.0 µV for a 50% increase in conductivity if we consider that the increase stopped at 500 Hz. Assuming that the increase continued linearly beyond 500 Hz, the values decreased to 28.9 and 27.7 µV for the 25% and 50% increase, respectively. While these latter results are substantially different from the results found assuming a fixed average conductivity, they stem from the assumption of an ever-increasing conductivity >500 Hz. This implies very high conductivity values for the highest frequencies contained in the spike signal, which are particularly important for determining the peak-to-peak spike amplitude. The conductivity in this frequency range is far beyond our experimentally probed frequency range, and we suspect that the conductivity values used are too high. Thus, the estimated peak-to-peak amplitude for this scenario is too low, and the deviation from the frequency-independent case is less than the computations imply.

**Figure 4.**
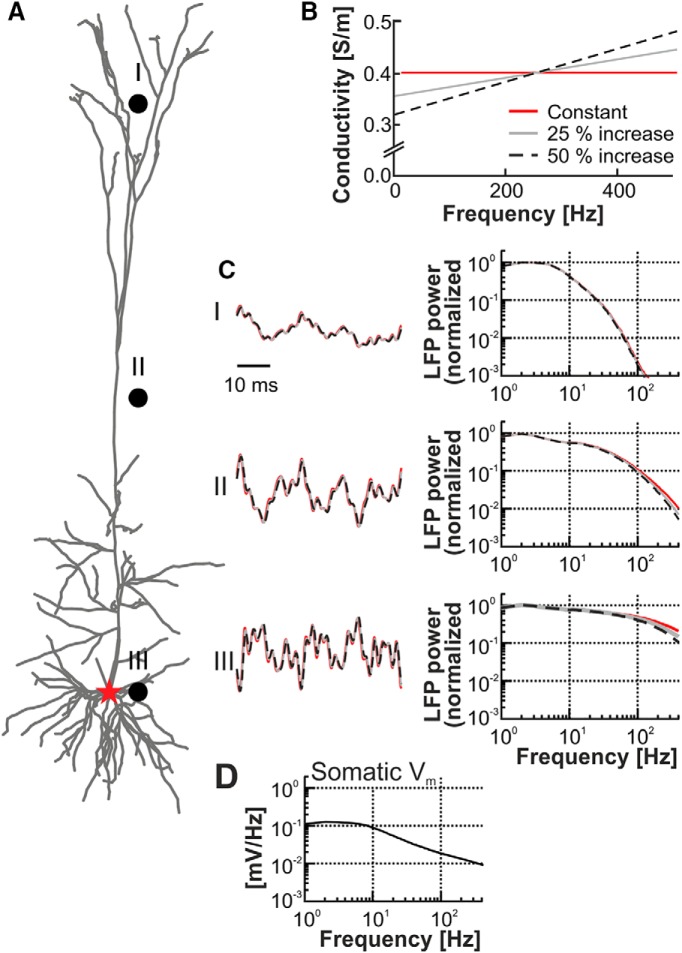
Effect of frequency-dependent conductivity on extracellular potentials arising from white noise input. ***A***, Somatodendritic reconstruction of a layer V pyramidal neuron and three simulated extracellular recording electrodes (black dots, layers I–III) and the location of simulated somatic white noise current input (red star). ***B***, Three different conductivity profiles, corresponding to a constant conductivity (red), a linear increase in conductivity from 5 to 500 Hz of 25% (gray) and 50% (black dashed). ***C***, Excerpts of normalized LFP signals recorded at the electrode points I–III for the different conductivity profiles in ***B*** and diagrams showing the respective normalized power spectral density of the LFPs. ***D***, Amplitude of somatic membrane potential response as a function of frequency in response to the white noise current input.

LFPs are often interpreted in terms of their power spectral density (PSD; i.e., the power of the signal at different frequencies), and it is therefore of interest to estimate the impact of a frequency-dependent conductivity of neural tissue on the LFP PSD. We injected white noise current (i.e., an input current with equal amplitude at all frequencies) into the soma of a cortical pyramidal cell model (Hay et al., 2011; Fig. 4*A*). As for the modeling of the consequences of a frequency-dependent increase of conductivity on signals originating from synaptic inputs and spikes, we calculated the resulting LFP at different positions along the apical dendrite, considering constant conductivity as well as an increase in the conductivity of 25% or 50% (Fig. 4*B*). At all recording electrode positions, a low-pass filter effect originating from intrinsic dendritic filtering was clearly visible (Fig. 4*C*; Lindén et al., 2010; Ness et al., 2016). The effect of an increase in the conductivity of 25% or 50% from 5 to 500 Hz had a negligible effect on the shape of the LFP PSD, compared with the strong shaping caused by the cable properties of the cellular membrane (i.e., the intrinsic dendritic filtering).

## Discussion

In acute *in vitro* brain slice preparations of the juvenile rat barrel cortex, we investigated the electrical conductivity of neural tissue in the submillimeter range by injecting extracellular low-amplitude current of different frequencies, ranging between 5 and 500 Hz. Our results indicate that tissue conductivity is not affected by either the position or the amplitude of the injected current, confirming that current injection within the extracellular medium can be successfully modeled as a point source, and that, as expected, neural tissue is a linear conductor for small, sub-nanoampere currents (Nunez and Srinivasan, 2006).

The main aim of our study was to investigate to what extent the conductivity of cortical brain tissue is affected by the frequency of electrophysiological neuronal signals generating the recorded extracellular potential (Gilja and Moore, 2007). We found statistically significant differences neither in the frequency dependence measured in cortical tissue and ACSF_C_ nor in the phase between tissue and saline recordings. Since saline is well known to be an ohmic medium, this suggests that cortical tissue is mainly an ohmic medium as well. If one assumes that the cause of the detected spurious frequency dependence in saline may have a reduced impact on the tissue recordings, then a relevant part of the detected 50% increase in conductivity from 5 to 500 Hz could indeed be caused by a real frequency dependence in neural tissue. Even in this case, the frequency dependence of cortical neural tissue would still be constrained to be a <50% increase in conductivity from 5 to 500 Hz. However, such a prominent tissue-specific contribution to detected frequency dependence appears unlikely because our data showed a zero phase shift. This implies that cortical neural tissue *in vitro* exhibits a similar frequency dependence at the 100 μm level with physiological current amplitudes, as it has previously been reported at the macroscopic scale *in vivo* (Nunez and Srinivasan, 2006; Logothetis et al., 2007; Wagner et al., 2014).

A 50% increase in the extracellular conductivity over 5 to 500 Hz would imply that the 500 Hz frequency component of the LFP is reduced by a factor of ∼0.66 compared with the 5 Hz component. As illustrated by biophysical modeling, such a 50% increase will have a negligible effect on the modeling and analysis of extracellular potentials originating from synaptic events or even spikes. In the latter case, the main effect of a higher conductivity at higher signal frequencies will mainly be a slight reduction of the amplitude of the sharp sodium peak. The observed frequency dependence is also far too small to account for purported 1/*f* power laws in the LFP power (Bédard et al., 2006; Bédard and Destexhe, 2009) that would require an average increase of the conductivity by ∼10,000% from 5 to 500 Hz.

**Figure 5.**
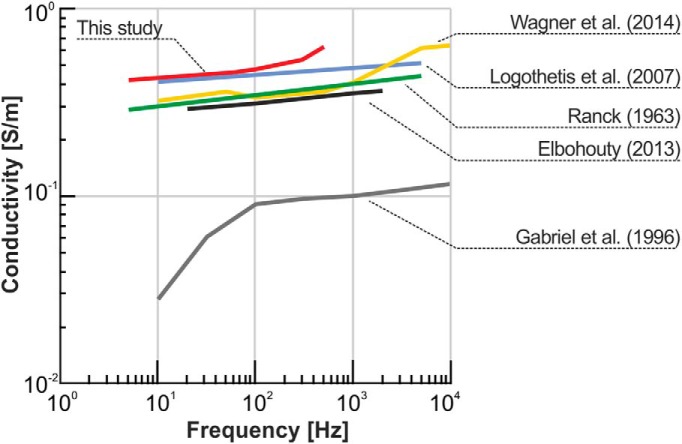
Literature review of reported conductivities in various species and experimental setups. Gabriel et al. (1996): data from bovine brains recorded with a two-electrode setup. Elbohouty (2013): data recorded *in vitro* in slices of mouse cerebral cortex with a two-electrode setup. Logothetis et al. (2007): data from monkey recorded with a four-electrode setup. The average conductivity value, 0.405 S/m, combined with the reported increase of ∼25%. Wagner et al. (2014): recordings from cat cerebral cortex with a two-electrode setup, which was similar to the setup used by Gabriel et al. (1996). Ranck (1963): data from rabbit brain.

In general, our results are qualitatively in good agreement with several previous *in vivo* and *in vitro* studies in brain tissue of various species (Fig. 5). Logothetis et al. (2007) found an increase of ∼25% in conductivity from 10 to 5000 Hz *in vivo* in monkeys, while Wagner et al. (2014) observed a similar frequency dependence for 10–1000 Hz in cats. Elbohouty (2013) measured the conductivity in slices of mouse cerebral cortex from 20 Hz to 2 MHz and also found a similar moderate increase between 20 and 1000 Hz. Dowrick et al. (2015) measured the impedance spectrum of rat brains in the frequency range from 10 to 3000 Hz, and, although they did not provide specific numbers for the measured conductivity, they reported a change in impedance of between 30% and 40% over the probed frequency range. In humans, Pfurtscheller and Cooper (1975) implemented current dipoles in the brain and reported that the signal attenuation in cortex and thorough the skull was independent of frequency.

The study of Gabriel et al. (1996) stands out as the results presented are unique in observing a strong frequency dependence of the conductivity <100 Hz (Fig. 5). However, in the study, the authors expressed concerns that the low-frequency values might be inaccurate due to inadequate correction for electrode polarization in their two-electrode setup. Note that Wagner et al., (2014) used similar recording equipment without observing an equally high frequency dependence <100 Hz. In addition to the strong frequency dependence, Gabriel et al. (1996) also reported a conductivity that approaches zero for low frequencies. This contrasts with the fact that neural tissue has a substantial amount of highly conductive CSF that should ensure that neural tissue has a substantial resistive component. The reasons for the discrepancy between the conductivities reported by Gabriel et al. (1996) and those of others are difficult to assess. However, based on the reported origin of the bovine brains tested by Gabriel et al. (1996), one can speculate that suboptimal neuronal tissue preservation may have resulted in cell swelling and degradation and, thus, possibly altered the composition and amount of the extracellular fluid.

Together, we conclude that for frequencies ranging between 5 and 500 Hz the frequency dependence of the electrical conductivity of cortical tissue is at most moderate, and for modeling and analysis purposes is largely negligible both *in vitro* and *in vivo*.

While our approach of injecting sinusoidal currents and measuring voltage responses can be extended to frequencies outside the present frequency range from 5 to 500 Hz, it should be noted that for frequencies less than some hertz, diffusion of ions in the extracellular space may play a role in setting up the electrical currents in situations comprising the emergence of large ionic concentration gradients. Such transient ionic gradients in the extracellular space could, for example, occur due to strong spatially restricted neuronal network activity resulting in the efflux of potassium from the cells and its local accumulation the extracellular fluid (Halnes et al., 2016). These diffusive electrical currents would, however, be independent of the imposed transmembrane currents (or in this case currents injected by the electrode) and, thus, would not affect the present measurement of the extracellular conductivity. However, based on measurements of transmembrane impedance, Gomes et al. (2015) suggested large imaginary components of the extracellular electrical conductivity with their origin in ion diffusion, even for frequencies as high as 1000 Hz. Their argument was based on the fact that models with a purely resistive extracellular medium could not account for their measured transmembrane impedance power spectra. Their modeling was performed using simplified stylized stick model neurons, however. With a biophysically more detailed multicompartmental neuron model (Hay et al., 2011), we obtain a qualitatively different power spectrum and observe a transmembrane impedance power spectrum that is generally in good agreement with the experimental findings of Gomes et al. (2015); compare our Fig. 4*D* with their Fig. 2B. On balance, we thus find that the overall evidence points to an essentially real (ohmic) extracellular conductivity with negligible effects from ionic diffusion in the frequency range between 5 and 500 Hz.

Previous reports have shown that the conductivity of brain tissue is anisotropic, especially if the underlying cytoarchitecture exhibits a strongly ordered organization of apical dendrites and/or fiber bundles (Nicholson and Freeman, 1975; Goto et al., 2010). Our experimental setup could in principle also be used to probe for anisotropy of the conductivity as the brain slice preparations of the barrel cortex we used for our experiments preserve the ordered organization of the apical dendrites of the pyramidal cells, which run orthogonally to the pia and in parallel with the tissue surface. In the same cortical region, Goto et al. (2010) found up to 50% higher conductivity along the primary axis of the apical dendrites of the large pyramidal cell compared with the lateral directions. In contrast to this, in our study, recording extracellular potentials via an MEA, we did not observe any anisotropy. In our study, an anisotropy in the neural tissue would emerge as different decays of signal amplitude with distance in the two directions of the MEA plane. This effect was not observed in our experimental data, which could be well fitted by a single distance decay (Fig. 1*C4* ). However, in exploring the effects of putative anisotropies in biophysical forward modeling data, a previous study by Ness et al. (2015) showed that MEA potentials are rather insensitive to anisotropies. Consequently, our approach using an MEA recording system does not allow us to draw strong conclusions about the anisotropic properties of tissue conductivity.

Our results are indicative of cortical tissue being frequency independent; however, a weak frequency dependence cannot be ruled out. Indeed, the overall trend of the results arising from various studies (Fig. 5) seems to imply a weak or modest increase in the conductivity between 5 and 1000 Hz. If this is the case, it would seem plausible that, based on biophysical properties, at lower frequencies the extracellular currents are conveyed by ions meandering between the largely insulating cells in the tissue (Peters et al., 2001), which is possibly enhanced by ionic currents passing through cells via open ion channels (Meffin et al., 2012, 2014). For frequencies of tens of hertz or more, one might expect that capacitive currents may additionally contribute to such neuron-crossing currents and thus provide a gradually increasing conductivity with frequency.

Nelson et al. (2008) and Nelson and Pouget (2010) argue that, under the assumption that the proper recording equipment is used, one does not need to worry about how the electrode properties affect the measured LFP. Our results, and most of the results of other studies, also indicate that the filtering properties of the tissue itself should be a minor factor in shaping the LFP. Thus, the interpretation of the LFP should focus on cell and network properties, without the added complication of electrode and tissue effects. As pointed out by Nelson and Pouget (2010), this might be one of the rare cases in neuroscience where what makes everything easier in terms of data interpretation is also likely to be true.

## Synthesis

Reviewing Editor: Upinder Bhalla, National Center for Biological Sciences Decisions are customarily a result of the Reviewing Editor and the peer reviewers coming together and discussing their recommendations until a consensus is reached. When revisions are invited, a fact-based synthesis statement explaining their decision and outlining what is needed to prepare a revision will be listed below. The following reviewer(s) agreed to reveal their identity: Felix Schürmann, Ulrich Hofmann.

The reviewers agreed that the authors have carried out a relevant study with

potentially useful contributions to the field.

1. They agreed that the authors should present their results more clearly

and indicate how robust they are.

2. The linkage between modeling and experiments could be strengthened.

In addition each of the reviewers had a number of specific points that the

authors should address.

------------------------------------------------------------

Reviewer 1.

The manuscript contributes to the somewhat elusive question on the electrical properties of cortical tissue, in particular, whether there are frequency-dependent effects that can affect the interpretation of signals such as the local field potential. While the study does not fully answer this question, it adds to the body of literature attesting no or only a small frequency dependence of the tissue impedance and it counter-balances results from a previous study (Gabriel 1996) showing a large frequency-dependence. Through modeling the study furthermore shows that the remaining frequency dependence is mostly negligible for the interpretation of LFP signals.

Comments:

- I commend the effort of the authors to bring more clarity to the discussion on the frequency dependence of tissue impedance and the role for the interpretation of LFP through experimental measurements and computational modeling; I think the authors make a useful contribution to the discussion - however, I was a little bit disappointed by the inconclusiveness of whether the impedance finally is mildly frequency dependent or not at all. While it doesn't change the overall conclusions of the study, in the part where the electrode polarization is studied, the authors set out to answer the question (saline solution should show EP if there is frequency dependence) but when it comes to the conclusion on the situation in tissue, the authors are more hesitant to be clear (at one point they say "at most moderate dependency", in the discussion they say "compatible with both", "overall trend seems to imply modest dependency". I don't know whether that comes from the fact that the authors do not fully trust their analysis as to where the EP occurs? So, either the authors present a more compelling analysis of their results that can discern clearly the contribution of the EP. Or the authors find a more consistent language throughout the manuscript that shows the limits of their data.

- along with this first request for a more clear positioning of the paper, comes a second: Through modeling, the authors show that even if there was a modest frequency dependence, for the interpretation of the signals this may not be so important. However, here, I think the authors may need to show some more modeling results (not only three simulated recording sites) to convince the reader that the results are robust in space. Also, as the authors cannot see evidence for anisotropy in the experimental recording, it may be good to show whether their modeling could similarly be used to estimate how a modest anisotropy would show up in the LFP signal interpretation. This way, the paper would address both aspects, frequency-dependence and space dependence, in a consistent prediction based on experimental data (non or moderate dependency). At the same time, it would bring some clarity on the robustness of the modeling results.

- The authors make the statement that by placing the patch electrode as far away from neuron somata as possible, intracellular signal propagation was prevented; can the authors support this with data? would it be a good idea to consider a control with TTX?

- when the authors model the effect of a frequency dependence of the conductance, they extend their study to beyond 500Hz. Why has the respective range not tested in experiment? it seems to weaken the conclusions a little bit?

Figures:

- overall, the quality of figures is improvable

- Figure 1: especially row C is not of sufficient quality; C1 is very dark; C2 is not in sufficient quality to interpret the the gray traces; C3 could have more explanation on the phase color scale (also, why is the hotspot off center to the location of the current injection when compared to C1?)

- Figure 2: I am not convinced that the figure is the most efficient presentation of the data: Essentially, panel C1 summarizes all panels of A and B efficiently and I am not convinced panels A and B add much to the interpretation; (the one outlier with higher conductivity that panels B shows furthermore goes uncommented)

- Figure 5: color choice of data series is essentially undecipherable

Minor:

- last paragraph of the introduction goes beyond scope of introduction (it summarizes results); authors may want to consider to remove/move those statements

- there are inconsistencies in the choice of British English (e.g. "modelling") and American English (e.g. "motorized") which should be corrected

- spelling mistakes "set up" line 564

- there are spurious "." or white spaces/missing white space, e.g. in lines 564 or line 529

- line 465: "hundredth" - I assume that is a mistake and should read "hundred"

-------------------------------------------------------

Reviewer 2

This study corroborates the known state of the art by measuring electrical conductivities in in vitro brain slices, utilizing MEA and 4 electrode setup. It is didactically not quite clear, why the authors elucidate measured effects by computational realistic models of a neuron under several conditions. That may be due to a slightly misleading title.

Statistics How can I judge the correctness? I can only evaluate their reasoning, assuming the calculations are correct. The presented values are originating from a set of measurements and as thus, it is reasonable to assume the statistical analysis is sound and correct - however: I did not re-evaluate their numbers.

Again, they do look reasonable, though

Comments to the Authors (Required) Why did you clip your max stimulation frequency at 500Hz - while stating the "less than a few thousand hertz" (l44) are the physiologically relevant frequencies?

l84: Please add the ethics approval number.

l91: The slice thickness was chosen to identify single somata and the stimulate in between - stay the results the same, when a thicker, say a 300-400µm slice, is used? Can you be sure that with a 200µm slice there is no shunting effect along the extra slice medium?

l102: Are the measured values all comparable at physiological temperatures?

l104: Did you test for slice viability at the end of the experiments? Your flow rate may cause hypoxia and thus a quick autolysis of tissue. Thus endangering the integrity of conductive pathways over time.

l129: You later describe "as far away as possible" (l363) - please do so already here.

l133-140: Please elaborate on the rationale of stimulation amplitude, since the input resistance picture holds only true for a patched cell - not for the extracellular stimulation you perform here.

l173: "directly" is an over-exaggeration here, I would say.

l188; You "reckon that the main polarization will happen at the silver-ball electrode". Two things here: Why does the metal wire providing the current source with the intra-patch medium not show any polarization - or at least can be ignored? How did you ensure, that the silver ball was pristine and perfect at each experiment? You didn't mention a cleaning procedure and from own experience silver surfaces are a lt easier disturbed than e.g. Pt.

Would a metal microwire show the same effects as compared to the patch-pipette? Why are Martinsen and Kalvøy so sure about the mechanism?

l484: You are dismissing here Gabriel's work quite easily(as in l54) - does your setup in 2-electrode configuration account for the factor of 10 in conductivity?

l463: Please reduce the high redundancy in the sentence: (about 50% over two orders of magnitude) - the reader has seen that several times by then.
